# Supplementary material for: Higher-order structures of the foot-and-mouth disease virus RNA-dependent RNA polymerase required for genome replication
Source: Commun Biol. 2022 Jan 17;5:61. doi: 10.1038/s42003-021-02989-z (PMC8764057; doi:10.1038/s42003-021-02989-z)
Supplement: Supplementary file 1 — Supplementary Information [file 42003_2021_2989_MOESM1_ESM.pdf]

Higher-order structures of the foot-and-mouth disease virus RNA-dependent RNA polymerase required for genome replication

Eleni-Anna Loundras, James Streetley, Morgan R. Herod, Rebecca F. Thompson, Mark Harris, David Bhella, Nicola J. Stonehouse

Supplemental data

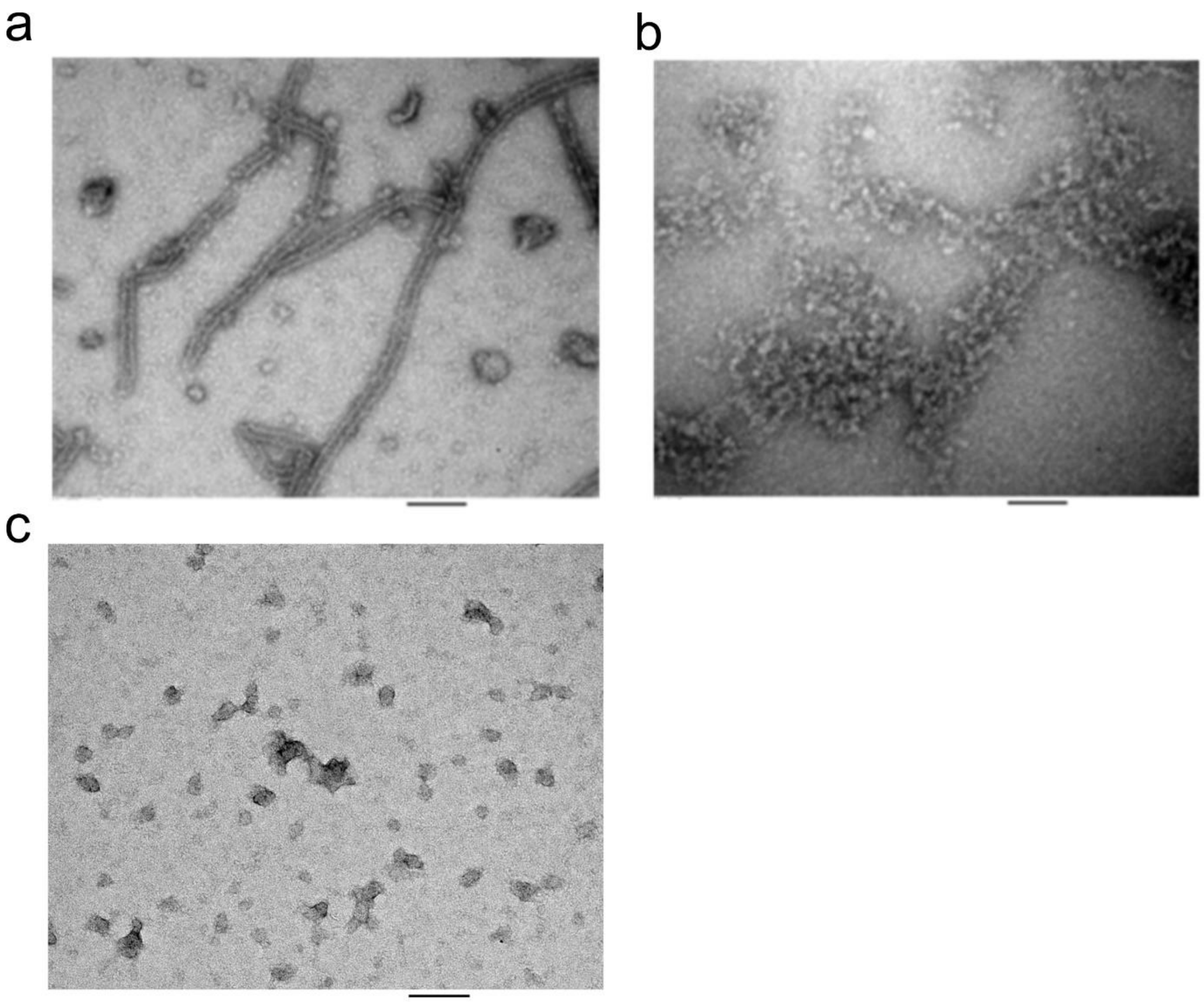

Fig. S1

Negative stain TEM of a sample from a polymerase activity assay in the presence of glutaraldehyde after 30-minute incubation. a) $3D^{pol}$-WT formed fibrils that were readily imaged. b) Imaging of RNase-treated WT fibrils did not show presence of fibrils. c) 3Dpol-GC216/7AA (in which the mutation is located in the RNA exit site) also did not show fibril formation. Scale bar 100 nm.

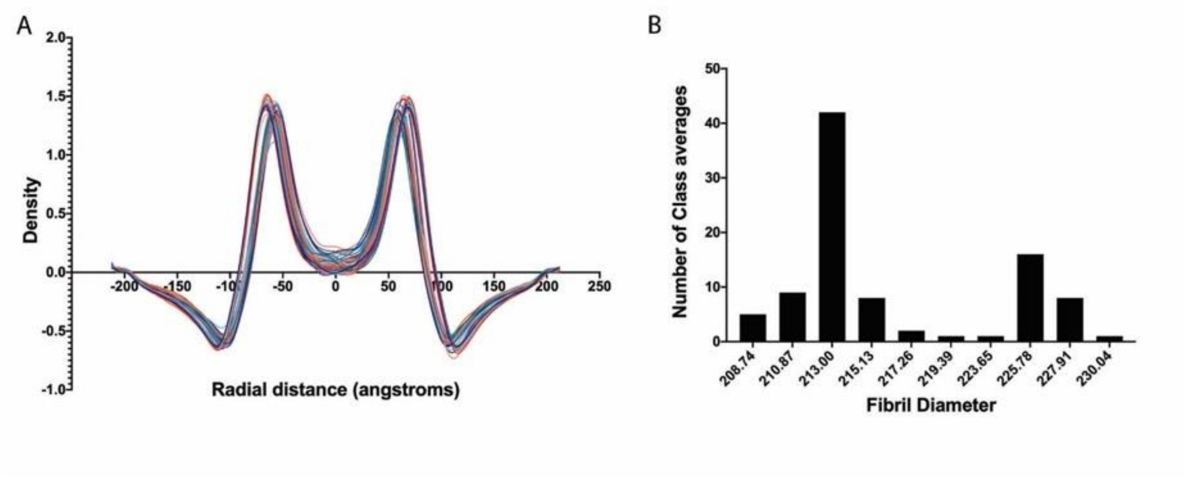

Fig. S2

a) Radial density profiles for 100 2D class averages calculated from ~250,000 fibril image sections. Each class average was projected along the helix axis, summing the density into a 1D profile. For ease of interpretation x-axis values are given as distances from the centreline of the 2D class average images, as an approximation of the helix axis position. b) To estimate the fibril diameters for each class average image, the distance between minima in each radial density plot was calculated, a histogram of these data shows a bimodal distribution of fibril diameters ranging between 20.8 and 23 nm.

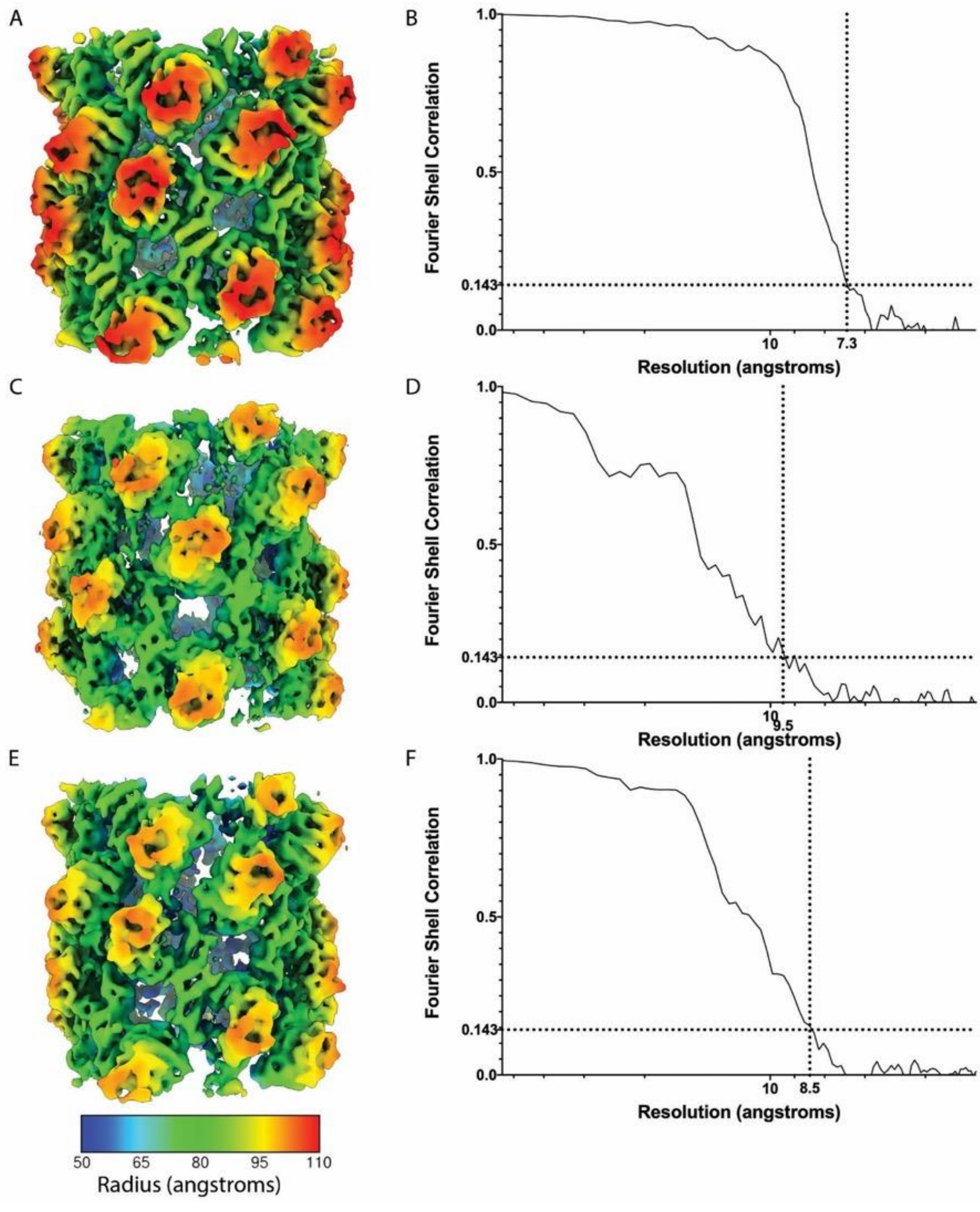
A
B
1.0
0.5
0.143
0.0
Fourier Shell Correlation
10
7.3
Resolution (angstroms)
C
D
1.0
0.5
0.143
0.0
Fourier Shell Correlation
10
9.5
Resolution (angstroms)
E
F
1.0
0.5
0.143
0.0
Fourier Shell Correlation
10
8.5
Resolution (angstroms)
50
65
80
95
110
Radius (angstroms)

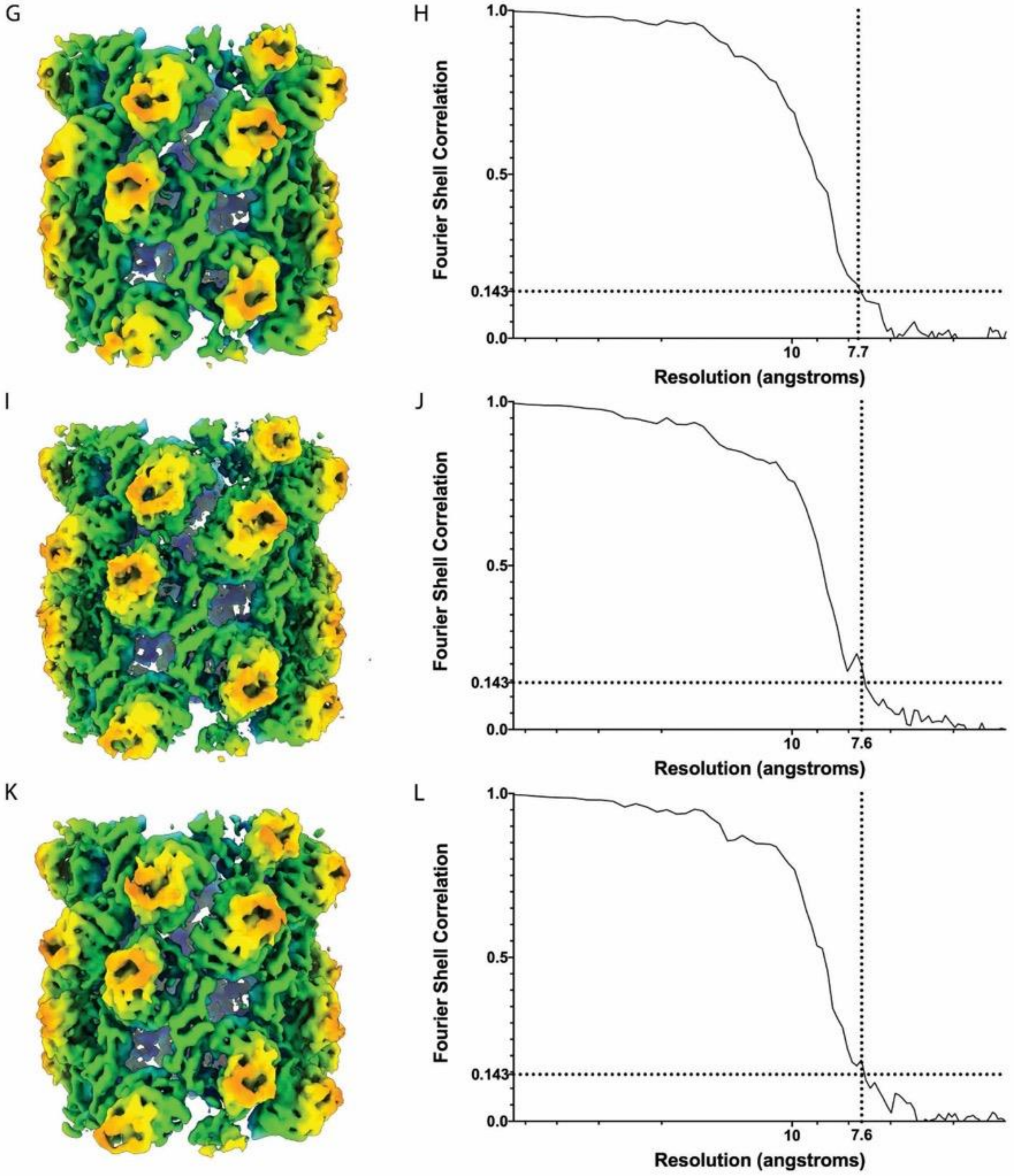
G
H
I
J
K
L
1.0
0.5
0.143
0.0
10
7.7
7.6
Fourier Shell Correlation
Resolution (angstroms)

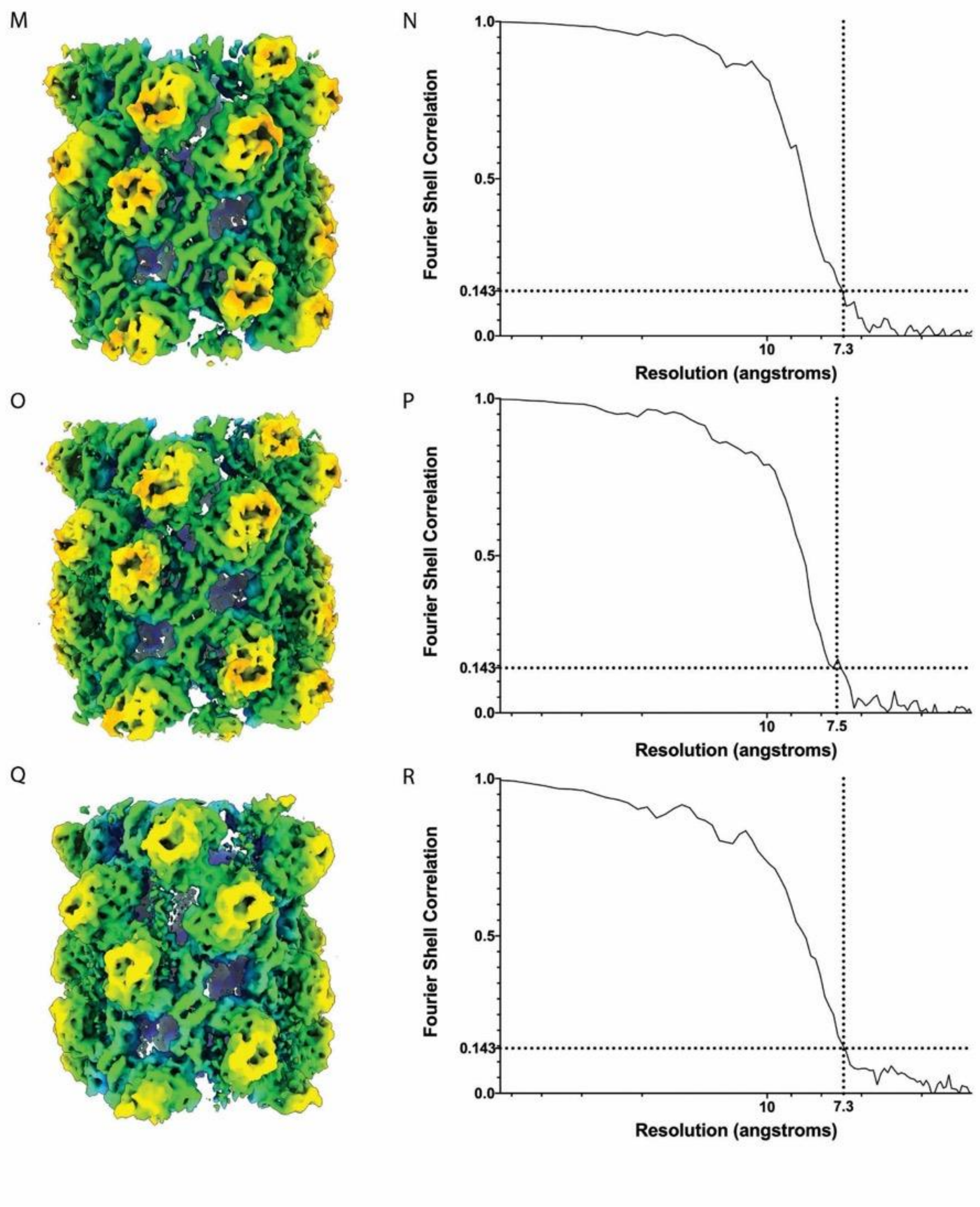
M
N
1.0
0.5
0.143
0.0
Fourier Shell Correlation
10
7.3
Resolution (angstroms)
O
P
1.0
0.5
0.143
0.0
Fourier Shell Correlation
10
7.5
Resolution (angstroms)
Q
R
1.0
0.5
0.143
0.0
Fourier Shell Correlation
10
7.3
Resolution (angstroms)

Fig. S3

Radial coloured 3D isosurface representations and Fourier shell correlation plots for the each cryo-EM 3D reconstruction. Resolution was estimated as the point at which correlation between two half-maps, calculated independently using Relion according to the 'gold-standard' method, drops below 0.143. (a, b) B1, (c, d) B2, (e, f) N3, (g, h) N4, (i, j) N5, (k, l) N6, (m, n) N7, (o, p) N8, (q, r) N9.

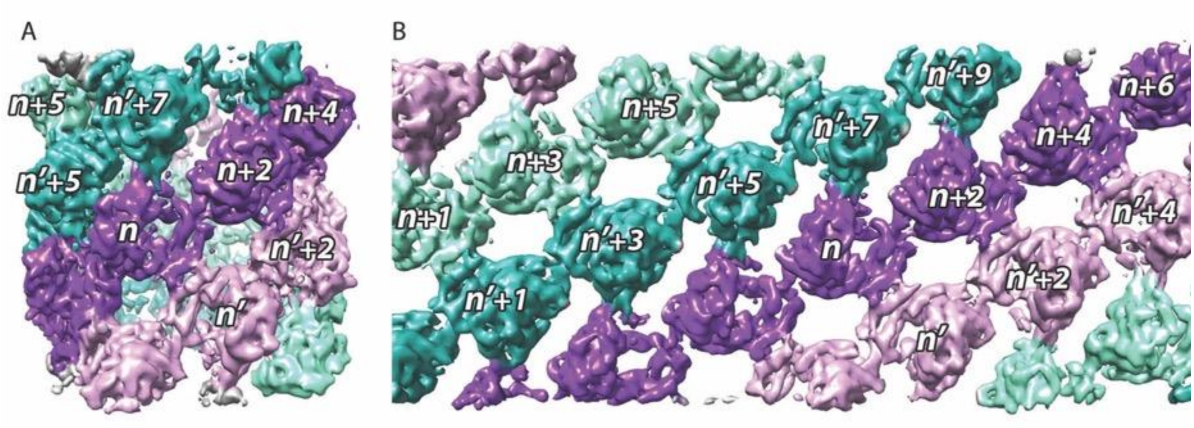

Fig. S4

The cryo-EM reconstruction for the tight/narrow fibril conformation N3 is shown coloured to highlight the two protofilaments present. Protofilament 1 is shown as purple/plum, while protofilament 2 is shown as aquamarine/light sea green. The notation used to describe interactions between $3D^{pol}$ is illustrated both on the reconstructed density map (a), and on a representation of the same map that has been unrolled to produce a planar array (b).

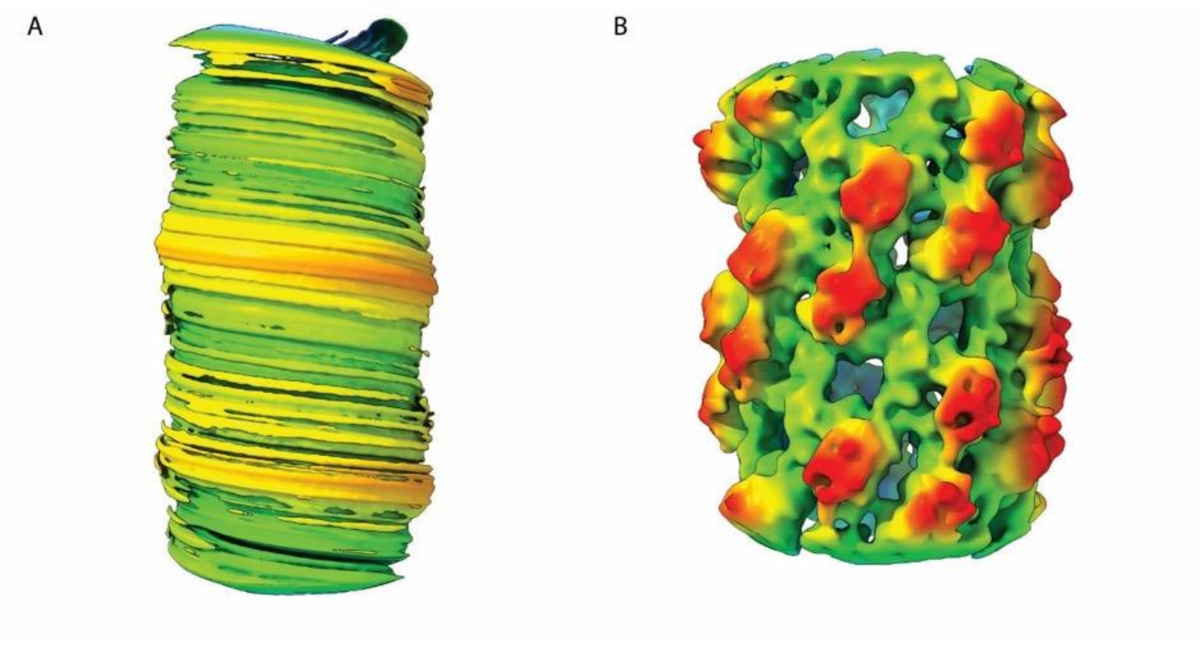

Fig. S5

Preliminary 3D reconstruction analysis of $3D^{pol}$ fibrils. A small dataset was collected on a JEOL 2200 cryomicroscope equipped with a Direct Electron DE20. An initial model was generated (a) and used as a starting point for helical reconstruction, which led to an interpretable density map (b).

Supplemental Table 1

A. Contact residues at the dimer interface

Conformation B1 *n*	Conformation B1 *n'*
Y394, G395, T396, F398, P436, Y455, R456, S457, Y459, L460, V463, V466, C467, D469, A470	Y394, G395, T396, F398, P436, Y455, R456, S457, Y459, L460, V463, V466, C467, D469, A470
Conformation B2 *n*	Conformation B2 *n'*
Y394, G395, T396, F398, P436, Y455, R456, S457, Y459, L460, V463, V466, C467, D469, A470	Y394, G395, T396, F398, P436, Y455, R456, S457, Y459, L460, V463, V466, C467, D469, A470
Conformation N3 *n*	Conformation N3 *n'*
Y394, G395, T396, P436, Y455, R456, S457, Y459, L460, V463, N464, C467, D469, A470	Y394, G395, T396, F398, P436, Y455, R456, S457, Y459, L460, V463, N464, C467, D469, A470
Conformation N4 *n*	Conformation N4 *n'*
Y394, G395, T396, F398, P436, Y455, R456, S457, Y459, L460, V463, C467, D469, A470	Y394, G395, T396, F398, P436, Y455, R456, S457, Y459, L460, V463, C467, D469, A470
Conformation N5 *n*	Conformation N5 *n'*
Y394, G395, T396, F398, P436, Y455, R456, S457, Y459, L460, V463, V466, C467, D469, A470	Y394, G395, T396, F398, P436, Y455, R456, S457, Y459, L460, V463, C467, D469, A470
Conformation N6 *n*	Conformation N6 *n'*
Y394, G395, T396, F398, P436, Y455, R456, S457, Y459, L460, V463, C467, D469, A470	Y394, G395, T396, F398, P436, Y455, R456, S457, Y459, L460, V463, V466, C467, D469, A470
Conformation N7 *n*	Conformation N7 *n'*
Y394, G395, T396, G397, F398, Y455, R456, S457, Y459, L460, V463, C467, D469, A470	Y394, G395, T396, F398, P436, Y455, R456, S457, Y459, L460, V463, V466, C467, D469, A470
Conformation N8 *n*	Conformation N8 *n'*
Y394, G395, T396, G397, F398, P436, Y455, R456, S457, Y459, L460, V463, V466, C467, D469, A470	Y394, G395, T396, G397, F398, P436, Y455, R456, S457, Y459, L460, V463, V466, C467, D469, A470
Conformation N9 *n*	Conformation N9 *n'*
Y394, G395, T396, G397, F398, P436, Y455, R456, S457, Y459, L460, V463, N464, C467, D469, A470	Y394, G395, T396, G397, F398, P436, Y455, R456, S457, Y459, L460, V463, N464, C467, D469, A470

B. Contact residues at the dimer-dimer interface

Conformation B1 *n*	Conformation B1 *n+2*
A22, P23, V25, A415, R416, R417, I420, P445, L449	L73, R76, H322, E324, G325, T330, Y346
Conformation B2 *n*	Conformation B2 *n+2*
A22, P23, V25, A415, R416, R417, I420, P445, L449	L73, R76, E324, G325, T330, Y346
Conformation N3 *n*	Conformation N3 *n+2*
A22, V25, A415, R416, R417, I420, L449	R76, E324, Y346
Conformation N4 *n*	Conformation N4 *n+2*
A22, V25, A415, R416, R417, I420, P445, L449, F450	L73, R76, E324, G325, T330, Y346
Conformation N5 *n*	Conformation N5 *n+2*
A22, P23, V25, A415, R416, R417, I420, P445, L449	L73, R76, H322, Y323, E324, G325, Y346, D347
Conformation N6 *n*	Conformation N6 *n+2*
A22, V25, A415, R416, R417, I420, P445, L449, F450	R76, E324, G325, Y346
Conformation N7 *n*	Conformation N7 *n+2*
A22, P23, V25, G28, A415, R416, R417, I420, P445, L449, F450	L73, R76, H322, Y323, E324, G325, T330, Y346, D347
Conformation N8 *n*	Conformation N8 *n+2*
A22, P23, V25, G28, A415, R416, R417, I420, P445, L449, F450	L73, R76, H322, Y323, E324, G325, D329 T330, Y346, D347
Conformation N9 *n*	Conformation N9 *n+2*
A22, V25, A415, R416, R417, I420, P445, L449, F450	L73, R76, E324, G325, T330, Y346

C. Contact residues at the protofilament interface

Conformation B1 *n*	Conformation 1 *n′+9*
E10, R12, H14, M16, N287	E10, R12, H14, M16, N287
Conformation B2 *n*	Conformation B2 *n′+7*
V4, D5, T6, R7, D8	V4, D5, T6, R7, D8
Conformation N3 *n*	Conformation N3 *n′+7*
E10, R12, H14, M16, N287	E10, R12, H14, M16, N287
Conformation N4 *n*	Conformation N4 *n′+7*
E10, R12, H14, M16, N287	E10, R12, H14, M16, N287
Conformation N5 *n*	Conformation N5 *n′+7*
E10, R12, H14, M16, N287	E10, R12, H14, M16, N287
Conformation N6 *n*	Conformation N6 *n′+7*
E10, R12, H14, M16, N287	E10, R12, H14, M16, N287
Conformation N7 *n*	Conformation N7 *n′+7*
R12, H14, M16, N287	R12, H14, M16, N287
Conformation N8 *n*	Conformation N8 *n′+5*
V4, K65	V4, K65
Conformation N8 *n*	Conformation N8 *n′+7*
R12, H14, M16, N287	R12, H14, M16, N287
Conformation N9 *n*	Conformation N9 *n′+5*
V4, D5, T6, R7, D8	V4, D5, T6, R7, D8

Tables to show amino-acid residues identified in contacts/clash analyses of interfaces (a) between monomers in a single dimeric subunit (dimer interface), (b) between dimers leading to the formation of ribbons of dimers (protofilaments) and (c) between protofilaments leading to the formation of fibrils. Contact residues common to all dimer and dimer-dimer interfaces are coloured red. Inter-protofilament contact residues common to tight forms are coloured orange, those common to open forms are coloured blue.
