# Supplementary material for: Higher-order structures of the foot-and-mouth disease virus RNA-dependent RNA polymerase required for genome replication
Source: Commun Biol. 2022 Jan 17;5:61. doi: 10.1038/s42003-021-02989-z (PMC8764057; doi:10.1038/s42003-021-02989-z)
Supplement: Supplementary file 2 — Description of Additional Supplementary Files [file 42003_2021_2989_MOESM2_ESM.pdf]

Description of Additional Supplementary Files

File name: Movie S1
Description: Movie to show nine fibril conformations determined by cryo-EM and helical reconstruction. Each conformation is shown as a radially coloured isosurface. Conformation N3 is shown as a transparent isosurface with a monomer of $3D^{pol}$ as a rainbow coloured ribbon diagram (timepoint 2.02). A second $3D^{pol}$ molecule is shown to illustrate the formation of dimers (timepoint 2.17). Protofilament one is shown as a ribbon of dimers coloured violet and thistle (timepoint 2.34). Protofilament two is shown coloured teal and aquamarine (timepoint 2.42).

Morphs are presented between conformations using solvent excluded surface representations. Conformations N3 to N8 show similar packing of protofilaments. Variation in fibril diameter is a result of protofilaments flexing/sliding. Narrow conformations have 8.4-8.57 dimers in each protofilament per helix turn. Morph of N3-N8 highlights the closing together of protofilaments resolved for the tight/narrow forms (timepoint 2.48 to 2.54). Conformation B1 also shows protofilaments packing tightly together, however in this conformation the two protofilaments are symmetric across the helix axis (C2 symmetry). Broad conformations have 9-9.2 dimers per turn in each protofilament. Morph of B1-N3 illustrate the difference between tight/narrow and tight/broad conformations (timepoint 2.58-3.02). For narrow and broad fibrils, reconstructions were calculated that showed a more open conformation - B2 and N9. Similar to the densely packed fibrils, these structures were formed of protofilaments of dimers. The contact interface between protofilaments was rather different however. Morph animations are intended to highlight how these open conformations differ from densely packed fibrils, showing how protofilaments slide against each other leading to opening of a cavity between the two ribbons and a further narrowing of the fibril (timepoints 1.45 to 1.50). Morphs between tight and open conformations (B1-B2) timepoint 3.06-3.11 (N3-N9) timepoint 3.16-3.23.

File name: Movie S2
Description: Movie to show protein-protein interactions that lead to the formation of FMDV $3D^{pol}$ fibrils. N3 is shown as a transparent isosurface with a pseudoatomic model of the fibril shown coloured to highlight the domain structure of the RNA dependent RNA polymerase; palm domain (plum), fingers domain (powder blue) and thumb domain (gold). Contacts/clashes at the dimer interface (timepoint 0.37), inter-dimer interface (timepoint 1.00) and inter-protofilament interface (timepoint 1.24) are shown as white dashed lines. Fibril N9 is shown as a transparent isosurface with fitted atomic models (timepoint 1.37), the inter-protofilament contact interface is highlighted (timepoint 2.08). The interface between protofilaments may involve the formation of an extended [illegible]-sheet, putative hydrogen bonds are indicated by blue dashed lines.

File name: Movie S3
Description: Movie to show cryo-EM density that is not assigned to protein by rigid-body fitting of PDB 2EC0, we hypothesise that density seen in the active site of $3D^{pol}$ indicates the presence of RNA within fibrils that may be at low-occupancy or poorly ordered; N3 is shown as a radial-coloured isosurface cryo-EM map. Putative RNA density in the active site is indicated by a white arrow (timepoint 0.28). A transparent map is shown with fitted atomic coordinates for both protein and nucleic acid, as determined by (38 – timepoint 0.34). The open fibril form N9 has a large finger of density that extrudes from the active site and extends between protofilaments. We interpret this as being RNA (timepoint 0.57).

File name: Supplementary Data 1

Description: Excel spreadsheets showing all data values, the calculated averages and error for figures 1a, 1b, 3bm 3d and 7a. Each data set is provided on a separate page, appropriately labelled.
