# Supplementary material for: Higher-order structures of the foot-and-mouth disease virus RNA-dependent RNA polymerase required for genome replication
Source: Commun Biol. 2022 Jan 17;5:61. doi: 10.1038/s42003-021-02989-z (PMC8764057; doi:10.1038/s42003-021-02989-z)
Supplement: Supplementary file 7 — Reporting Summary [file 42003_2021_2989_MOESM7_ESM.pdf]

nature research

Corresponding author(s): *Double-blind peer review submissions: write DBPR and your manuscript number here instead of author names.*

Last updated by author(s): *YYYY-MM-DD*

Reporting Summary

Nature Research wishes to improve the reproducibility of the work that we publish. This form provides structure for consistency and transparency in reporting. For further information on Nature Research policies, see our Editorial Policies and the Editorial Policy Checklist.

Statistics

For all statistical analyses, confirm that the following items are present in the figure legend, table legend, main text, or Methods section.

n/a	Confirmed	
☐	☒	The exact sample size (*n*) for each experimental group/condition, given as a discrete number and unit of measurement
☐	☒	A statement on whether measurements were taken from distinct samples or whether the same sample was measured repeatedly
☒	☐	The statistical test(s) used AND whether they are one- or two-sided<br>*Only common tests should be described solely by name; describe more complex techniques in the Methods section.*
☒	☐	A description of all covariates tested
☒	☐	A description of any assumptions or corrections, such as tests of normality and adjustment for multiple comparisons
☒	☐	A full description of the statistical parameters including central tendency (e.g. means) or other basic estimates (e.g. regression coefficient) AND variation (e.g. standard deviation) or associated estimates of uncertainty (e.g. confidence intervals)
☒	☐	For null hypothesis testing, the test statistic (e.g. *F*, *t*, *r*) with confidence intervals, effect sizes, degrees of freedom and *P* value noted<br>*Give P values as exact values whenever suitable.*
☒	☐	For Bayesian analysis, information on the choice of priors and Markov chain Monte Carlo settings
☒	☐	For hierarchical and complex designs, identification of the appropriate level for tests and full reporting of outcomes
☒	☐	Estimates of effect sizes (e.g. Cohen's *d*, Pearson's *r*), indicating how they were calculated

Our web collection on statistics for biologists contains articles on many of the points above.

Software and code

Policy information about availability of computer code

Data collection	Data were collected using Thermo Fisher Scientific EPU
Data analysis	Data analysis for Cryo-EM data: Initial model generation was performed using SPIDER (Frank, J., Radermacher, M., Penczek, P., Zhu, J., Li, Y., Ladjadj, M. and Leith, A. 1996. SPIDER and WEB: processing and visualization of images in 3D electron microscopy and related fields. Journal of structural biology. 116(1), pp.190–9.), Motion correction was performed using Motioncor2 with dose-weighting (Zheng, S.Q., Palovcak, E., Armache, J.-P., Verba, K.A., Cheng, Y. and Agard, D.A. 2017. MotionCor2: anisotropic correction of beam-induced motion for improved cryo-electron microscopy. Nature methods. 14(4), pp.331–332.). CTF estimation was performed using GCTF (Zhang, K. 2016. Gctf: Real-time CTF determination and correction. Journal of Structural Biology. 193(1), pp.1–12.). Helical reconstruction was performed using Relion 3.0 followed by 3.1 (Scheres, S.H.W. 2012. RELION: Implementation of a Bayesian approach to cryo-EM structure determination. Journal of Structural Biology. 180(3), pp.519–530, He, S. and Scheres, S.H.W. 2017. Helical reconstruction in RELION. Journal of structural biology. 198(3), pp.163–176). 2D radial density analysis was performed using SPIDER and BSOFT (Heymann, J.B. 2001. Bsoft: image and molecular processing in electron microscopy. Journal of structural biology. 133(2–3), pp.156–69.) and with GraphPad Prism v. 8. Reconstructions were visualised using UCSF Chimera (Pettersen, E.F., Goddard, T.D., Huang, C.C., Couch, G.S., Greenblatt, D.M., Meng, E.C. and Ferrin, T.E. 2004. UCSF Chimera--a visualization system for exploratory research and analysis. Journal of computational chemistry. 25(13), pp.1605–12.) and UCSF ChimeraX (Goddard, T.D., Huang, C.C., Meng, E.C., Pettersen, E.F., Couch, G.S., Morris, J.H. and Ferrin, T.E. 2018. UCSF ChimeraX: Meeting modern challenges in visualization and analysis. Protein science : a publication of the Protein Society. 27(1), pp.14–25.)

For manuscripts utilizing custom algorithms or software that are central to the research but not yet described in published literature, software must be made available to editors and reviewers. We strongly encourage code deposition in a community repository (e.g. GitHub). See the Nature Research guidelines for submitting code & software for further information.

Data

Policy information about availability of data

All manuscripts must include a data availability statement. This statement should provide the following information, where applicable:

- Accession codes, unique identifiers, or web links for publicly available datasets
- A list of figures that have associated raw data
- A description of any restrictions on data availability

CryoEM maps have been deposited in the EM-databank (https://www.ebi.ac.uk/pdbe/emdb/) with the accession numbers (B1) EMD-12093, (B2) EMD-12094, (N3) EMD-12096, (N4) EMD-12097, (N5) EMD-12098, (N6) EMD-12099, (N7) EMD-12100, (N8) EMD-12101, (N9) EMD-12102. The raw micrograph movies are deposited in the EMPIAR databank with accession number 10602.

Field-specific reporting

Please select the one below that is the best fit for your research. If you are not sure, read the appropriate sections before making your selection.

☒ Life sciences ☐ Behavioural & social sciences ☐ Ecological, evolutionary & environmental sciences

For a reference copy of the document with all sections, see nature.com/documents/nr-reporting-summary-flat.pdf

Life sciences study design

All studies must disclose on these points even when the disclosure is negative.

Sample size	*Describe how sample size was determined, detailing any statistical methods used to predetermine sample size OR if no sample-size calculation was performed, describe how sample sizes were chosen and provide a rationale for why these sample sizes are sufficient.*
Data exclusions	No data was excluded from the biochemical analyses.
Replication	All biochemical experimental assays were performed at least three different times using complete biological repeats. If more repeats were undertaken these are noted.
Randomization	Negative controls using enzymatically inactive protein were included.
Blinding	*Describe whether the investigators were blinded to group allocation during data collection and/or analysis. If blinding was not possible, describe why OR explain why blinding was not relevant to your study.*

Reporting for specific materials, systems and methods

We require information from authors about some types of materials, experimental systems and methods used in many studies. Here, indicate whether each material, system or method listed is relevant to your study. If you are not sure if a list item applies to your research, read the appropriate section before selecting a response.

Materials & experimental systems

n/a	Involved in the study	
☐	☒	Antibodies
☐	☒	Eukaryotic cell lines
☒	☐	Palaeontology and archaeology
☒	☐	Animals and other organisms
☒	☐	Human research participants
☒	☐	Clinical data
☒	☐	Dual use research of concern

Methods

n/a	Involved in the study	
☒	☐	ChIP-seq
☒	☐	Flow cytometry
☒	☐	MRI-based neuroimaging

Antibodies

Antibodies used	Primary rabbit anti-3D 397 polyclonal antibody, secondary anti-rabbit HRP (sigma-aldrich)
Validation	*Describe the validation of each primary antibody for the species and application, noting any validation statements on the manufacturer's website, relevant citations, antibody profiles in online databases, or data provided in the manuscript.*

Eukaryotic cell lines

Policy information about cell lines

Cell line source(s)	BHK-21 baby hamster kidney fibroblast cell line from ATCC
Authentication	Cell lines were not authenticated
Mycoplasma contamination	Cell lines were tested for mycoplasma contamination by PCR
Commonly misidentified lines (See ICLAC register)	*Name any commonly misidentified cell lines used in the study and provide a rationale for their use.*
